# Na^+^/K^+^-ATPase: expression and regulation at the maternal-fetal interface during pregnancy in pigs

**DOI:** 10.5713/ab.260474

**Published:** 2026-07-27

**Authors:** Yohan Choi, Eunhyeok Choi, Heewon Seo, Seonghyun Kim, Yugyeong Cheon, Soohyung Lee, Inkyu Yoo, Hakhyun Ka

**Affiliations:** 1 Division of Biological Science and Technology and Basic Science Research Institute, Yonsei University, Wonju, Korea; 2 Department of Obstetrics and Gynecology, University of Kentucky College of Medicine, Lexington, KY, USA; 3 Department of Animal and Avian Sciences, College of Agriculture and Natural Resources, University of Maryland, College Park, MD, USA

**Keywords:** Endometrium, Na^+^/K^+^-ATPase, Pig, Pregnancy

## Abstract

**Objective:**

The establishment and maintenance of pregnancy require well-coordinated interactions between the endometrium and the conceptus involving cellular and secretory components. Na^+^/K^+^-ATPase, composed of alpha, beta, and Phe-X-Tyr-Asp (FXYD) subunits, plays important roles in maintaining Na^+^/K^+^ flux and mediating nutrient transport in the cell. Although the importance of Na^+^/K^+^-ATPase during pregnancy has been shown in some species, the expression, regulation, and function of Na^+^/K^+^-ATPase subunits at the maternal–conceptus interface remains poorly understood.

**Methods:**

We obtained endometrial tissues during the estrous cycle and pregnancy, conceptus tissues during early pregnancy, and chorioallantoic tissues from mid to late pregnancy, and analyzed the expression of Na^+^/K^+^-ATPase subunits in pigs. The effects of steroid hormones, cytokines, and A23187, a calcium ionophore, on the expression of Na^+^/K^+^-ATPase subunits were examined in endometrial explant cultures.

**Results:**

Several Na^+^/K^+^-ATPase subunits in the endometrium changed dynamically during pregnancy, with some subunits having higher expression during early pregnancy than during the estrous cycle. Endometrial and chorionic epithelial cells were the primary sites of expression for Na^+^/K^+^-ATPase subunits. Conceptus-derived estrogen, interleukin-1β and/or interferon-γ, as well as A23187 all increased the expression of many subunits. Na^+^/K^+^-ATPase subunits were also expressed in conceptuses during early pregnancy and in chorioallantoic tissues between mid to term pregnancy.

**Conclusion:**

Na^+^/K^+^-ATPase subunits expressed in endometrial, conceptus, and chorioallantoic tissues may play important roles in maintaining membrane potential and modulating nutrient transport in endometrial and conceptus tissues to ensure the successful establishment and maintenance of pregnancy in pigs.

## INTRODUCTION

Nutrient transport at the maternal-conceptus interface is an essential process for the successful establishment and maintenance of pregnancy. In species that form a true epitheliochorial-type placenta, including pigs, the maternal endometrial epithelial cells and placental chorionic epithelial (CE) cells at the attachment sites maintain intact throughout pregnancy [1]. In humans and rodents, which form a hemochorial type of placenta, maternal blood comes in direct contact with the fetal CE cells by invasion of trophoblast cells (hemochorial placentation) [1], which is efficient for nutrient transport and gas exchange from the maternal blood to the embryo/fetus through the placenta [2]. Regardless of placentation type, inappropriate nutrient transport may cause abnormal fetal growth, intrauterine growth restriction, and other potential pregnancy failures [3]. A variety of nutrient transporters play critical roles in mediating transport of nutrients at the maternal-conceptus interface in mammals, including glucose, amino acids, and calcium ions [4–7]. In pigs, many nutrient transporters such as glucose transporters (solute carrier 2A superfamily, SLC2As; SLC5As), amino acid transporters (SLC1As; SLC6As; SLC7As; SLC38As), and calcium transporters (plasma membrane calcium ATPases, ATP2Bs; SLC8As; SLC24As; and transient receptor potential cation channel subfamily V member 6) are expressed in the endometrium during the estrous cycle and at the maternal-conceptus interface during pregnancy [8–12].

Na^+^/K^+^-ATPase, an energy-transducing ion pump, is responsible for the maintenance of high concentrations of potassium ions but low concentrations of sodium ions in the cell by transport of these ions across the plasma membrane, thereby participating in a variety of physiological processes including maintenance of the cell membrane potential, osmolarity, and signal transduction [13,14]. Na^+^/K^+^-ATPase is comprised of three subunits, alpha (α), beta (β), and Phe-X-Tyr-Asp (FXYD) protein subunits. Catalytic α subunit functions as a transporter aiding opposite movement of sodium and potassium ions and also acts as an ATP hydrolyzing enzyme and an ouabain binding receptor [13–15]. There are four isoforms of the α subunit (ATP1A1-4), and they exhibit different enzymatic properties. ATP1A1 is ubiquitously expressed in nearly every tissue, but other isoforms are tissue-specific. Three isoforms of the N-glycosylated β subunit, ATP1B1-3, act as essential chaperones for α subunit trafficking to the plasma membrane. Four α and three β subunits are potentially able to assemble 12 differential isozymes with distinct functional properties, and these two subunits are sufficient to enable minimal function of Na^+^/K^+^-ATPase [13,14]. The FXYD family, composed of seven isoforms, is an auxiliary protein characterized by an FXYD motif, two conserved glycine residues, and a serine residue [15]. FXYD proteins finely regulate the apparent affinity of Na^+^/K^+^-ATPase to Na^+^ and K^+^ ions and stabilize Na^+^/K^+^-ATPase by protecting against thermal inactivation of Na^+^/K^+^-ATPase. The FXYD family is widely distributed in mammalian tissues [13–15].

In addition to maintaining sodium and potassium ion concentrations in the cell, Na^+^/K^+^-ATPase of the cell surface is also involved in the activities of nutrient transporters in endometrial and placental tissues by regulating the movement of sodium ions across the plasma membrane [16,17]. Na^+^/K^+^-ATPases participate in the transport of nutrients, including glucose, amino acids, and calcium ions, by interacting with these nutrient transporters [18–21]. Na^+^/K^+^-ATPase and glucose and amino acid transporters are expressed in the endometrium during the estrous cycle and pregnancy in various species, including rodents, humans, ruminants, and pigs [4,6–9,11,12,16,22,23]. These findings suggest that Na^+^/K^+^-ATPase plays important roles in the establishment and maintenance of pregnancy by regulating the cellular membrane potential, cell signaling, and nutrient transports.

Although Na^+^/K^+^-ATPase contributes to implantation, placentation, and fetal development by regulating nutrient transport at the maternal-conceptus interface during pregnancy in several species, the expression, regulation, and function of Na^+^/K^+^-ATPase subunits at the maternal-conceptus interface are not well understood in pigs. Therefore, in this study we determined the expression, localization, and regulation of Na^+^/K^+^-ATPase subunits, *ATP1As*, *ATP1Bs*, and *FXYDs*, in the endometrium during the estrous cycle and in the endometrial, conceptus, and chorioallantoic tissues during pregnancy in pigs.

## MATERIALS AND METHODS

### Animals and tissue preparation

Sexually mature Landrace and Yorkshire crossbred female pigs of similar age (6 to 8 months) and weight (100 to 120 kg) were observed daily for estrous behavior with intact males and assigned randomly to either cyclic or pregnant status groups. Gilts assigned to the pregnant group were artificially inseminated with fresh boar semen at the onset of estrus (Day 0) and 12 h later. The reproductive tracts of gilts were obtained immediately after slaughter on Days 12 (n = 5) and 15 (n = 4) of the estrous cycle and Days 10 (n = 4), 12 (n = 6), 15 (n = 6), 30 (n = 4), 60 (n = 6), 90 (n = 6), and 114 (n = 4) of pregnancy. Pregnancy was confirmed by the presence of normal spherical or filamentous conceptuses in uterine flushings on Days 10, 12, or 15 and by the presence of embryos and placentas on the later days of pregnancy. Uterine flushings were obtained by introducing and recovering 50 mL of phosphate-buffered saline (PBS, pH 7.4) from the uterus (25 mL/uterine horn). Chorioallantoic tissues were obtained on Days 30 (n = 4), 60 (n = 5), 90 (n = 4), and 114 (n = 4) of pregnancy. Endometrial tissues, which were dissected free of the myometrium and collected from the middle portion of each uterine horn, conceptuses obtained by uterine flushing and washed with PBS (pH 7.4), and chorioallantoic tissues were snap-frozen in liquid nitrogen and stored at –80°C prior to RNA extraction. For *in situ* hybridization analyses, cross-sections of the endometrium were fixed in 4% paraformaldehyde in PBS (pH 7.4) for 24 h and then embedded in paraffin, as previously described [24].

### Endometrial explant cultures

To determine the effect of steroid hormones interleukin-1β (IL1B), and interferon-γ (IFNG), which are produced by implanting porcine conceptuses, on endometrial Na^+^/K^+^-ATPase subunit expression, endometrial explant tissues from gilts on Day 12 of the estrous cycle were dissected from the myometrium and placed into warm phenol red-free Dulbecco’s modified Eagle’s medium/F-12 culture medium (DMEM/F-12; Sigma-Aldrich) containing penicillin G (100 IU/mL) and streptomycin (0.1 mg/mL), as previously described [24,25]. To determine the effect of steroid hormones on endometrial Na^+^/K^+^-ATPase subunit expression, endometrial explants were cultured with rocking immediately after mincing in the presence of ethanol (control), estradiol-17β (E2; 10 ng/mL, Sigma-Aldrich), progesterone (P4; 30 ng/mL; Sigma-Aldrich), P4+E2, P4+E2+ICI182,780 (ICI; an estrogen receptor antagonist; 200 ng/mL; Tocris Bioscience), or P4+E2+RU486 (RU, a progesterone receptor antagonist, 30 ng/mL; Sigma-Aldrich) for 24 h in an atmosphere of 5% CO_2_ in air at 37°C. To determine the effect of IL1B on endometrial Na^+^/K^+^-ATPase subunit expression, explant tissues were treated with 0, 1, 10, or 100 ng/mL IL1B (Sigma-Aldrich) in the presence of both E2 (10 ng/mL) and P4 (30 ng/mL at 37°C for 24 h). To determine the effect of IFNG on endometrial Na^+^/K^+^-ATPase subunit expression, endometrial explants were cultured with rocking in the presence of E2 (10 ng/mL), P4 (30 ng/mL), and IL1B (10 ng/mL) for 24 h in an atmosphere of 5% CO_2_ in air at 37^o^C and then for an additional 24 h with 0, 1, 10, or 100 ng/mL of IFNG (R&D Systems) in the presence of E2 (10 ng/mL), P4 (30 ng/mL), and IL1B (10 ng/mL). The doses of hormones and cytokines used were based on previous studies [26–28]. Explant tissues were then harvested, and total RNA was extracted for real-time reverse transcription-polymerase chain reaction (RT-PCR) to determine the expression levels of Na^+^/K^+^-ATPase subunit mRNAs. These experiments were conducted using the endometria from three gilts on Day 12 of the estrous cycle, and treatments were performed in triplicate using tissues obtained from each of the three gilts.

### Total RNA extraction, reverse transcription-polymerase chain reaction, and cloning of Na^+^/K^+^-ATPase subunit complementary DNAs

Total RNA was extracted from endometrial, conceptus, and chorioallantoic tissues using TRIzol reagent (Invitrogen) according to the manufacturer’s recommendations. The quantity of RNA was assessed spectrophotometrically, and RNA integrity was validated following electrophoresis in a 1% agarose gel. Four micrograms of total RNA were treated with DNase I (Promega) and reverse transcribed using SuperScript II reverse transcriptase (Invitrogen) to obtain complementary DNA (cDNA). cDNA templates were then diluted 1:4 with nuclease-free water and amplified by PCR using Taq polymerase (Takara Bio) and specific primers based on porcine mRNA sequences for Na^+^/K^+^-ATPase subunits. PCR conditions, sequences of primer pairs for Na^+^/K^+^-ATPase subunits, and expected product sizes are listed in Table 1. PCR products were separated on 2% agarose gels and visualized by ethidium bromide staining. The identity of each amplified PCR product was verified by sequence analysis after being cloned into the TOPOII vector (Invitrogen).

### Quantitative real-time reverse transcription-polymerase chain reaction

To analyze the mRNA levels of Na^+^/K^+^-ATPase subunits in endometrial and chorioallantoic tissues, real-time RT-PCR was performed using a StepOnePlus system (Applied Biosystems) with the SYBR Green method. cDNAs were synthesized from 4 μg of total RNA isolated from different uterine endometrial tissues and cultured cells and treated with DNase I (Promega). Newly synthesized cDNAs (total volume of 21 μL) were diluted 1:4 with nuclease-free water and then used for PCR. Power SYBR Green PCR master mix (Applied Biosystems) was used for PCR reactions. The final reaction volume of 20 μL comprised 2 μL of cDNA, 10 μL of 2× master mix, 2 μL of each primer (100 nM), and 4 μL of ddH_2_O. The PCR conditions, sequences of primer pairs used for Na^+^/K^+^-ATPase subunits and expected product sizes are listed in Table 1. The primer efficiency was in the 90%–110% range. The results are reported as expression relative to the level detected on Day 12 of the estrous cycle for endometrial tissues, Day 30 of pregnancy for chorioallantoic tissues, and the control group for endometrial explant tissues after normalizing the transcript amounts to the endogenous porcine ribosomal protein L7 (*RPL7*), TATA-binding protein (*TBP*), and ubiquitin B (*UBB*) controls using the 2^−ΔΔCT^ method [29].

### Nonradioactive *in situ* hybridization

To determine the type(s) of porcine endometrial cells expressing Na^+^/K^+^-ATPase subunits mRNA, nonradioactive *in situ* hybridization was performed as previously described [30], with some modifications. Sections (5 μm thick) were rehydrated through successive immersions in xylene, 100% ethanol, 95% ethanol, diethylpyrocarbonate (DEPC)-treated water, and DEPC-treated PBS. Tissue sections were boiled in citrate buffer (pH 6.0) for 10 min. After being washed in DEPC-treated PBS, the sections were digested using 5 μg/mL proteinase K (Sigma-Aldrich) in TE (100 mM Tris-HCl, 50 mM EDTA, pH 7.5) at 37°C. After fixation in 4% paraformaldehyde, the tissue sections were incubated twice in PBS containing 0.1% active DEPC for 15 min each time and equilibrated in 5× saline sodium citrate (SSC) buffer for 15 min. The sections were prehybridized for 2 h at 68°C in a hybridization mix (50% formamide, 5X SSC, 500 μg/mL herring sperm DNA, and 250 μg/mL yeast tRNA). Sense and antisense riboprobes for Na^+^/K^+^-ATPase subunits were generated using partial cDNAs cloned into pCRII vectors by linearizing them with the appropriate restriction enzymes and labeling them with digoxigenin (DIG)-UTP using a DIG RNA labeling kit (Roche). The probes were denatured for 5 min at 80°C and added to the hybridization mix.

The hybridization reaction was carried out overnight at 68°C. Prehybridization and hybridization reactions were performed in a box saturated with a 5× SSC 50% formamide solution to prevent evaporation without coverslips. After hybridization, sections were washed for 30 min in 2× SSC at room temperature, 1 h in 2× SSC at 65°C, and 1 h in 0.1× SSC at 65°C. Probes bound to each section were detected immunologically using sheep anti-DIG Fab fragments covalently coupled to alkaline phosphatase and nitro blue tetrazolium chloride/5-bromo-4-chloro-3-indolyl phosphate (toluidine salt) (Roche) as a chromogenic substrate, according to the manufacturer’s protocol. The tissue sections were then washed in water and coverslipped. Microscopic images were captured using an Eclipse TE2000-U microscope (Nikon) and processed with Adobe Photoshop software (Adobe Systems).

### Statistical analysis

Data from real-time RT-PCR were subjected to analysis of variance using the General Linear Models procedures in SAS. As sources of variation, the model included day, pregnancy status (cyclic or pregnant, Days 12 and 15 post-estrus), and their interactions to evaluate steady-state levels of Na^+^/K^+^-ATPase subunit expression. Data from real-time RT-PCR were analyzed using least squares regression analyses to evaluate the effects of pregnancy day on endometrial tissue (Days 10, 12, 15, 30, 60, 90, and 114) and chorioallantoic tissue (Days 30, 60, 90, and 114), as well as the effects of IL1B and IFNG doses on explant cultures. Data from real-time RT-PCR to assess the effects of steroid hormones on explant cultures were analyzed using preplanned orthogonal contrasts (control vs. E2; control vs. P4; P4 vs. P4+E2; P4+E2 vs. P4+E2+ICI; and P4+E2 vs. P4+E2+RU). Before analysis, all data were tested for normality and homogeneity of variances, and log transformation was performed when necessary. Data are presented as means with standard errors of the mean. p-values less than 0.05 were considered significant, and p-values 0.05–0.10 were considered to indicate trends toward significance.

## RESULTS

### Expression of Na^+^/K^+^-ATPase subunits in the endometrium during the estrous cycle and pregnancy in pigs

**Figure 1:**
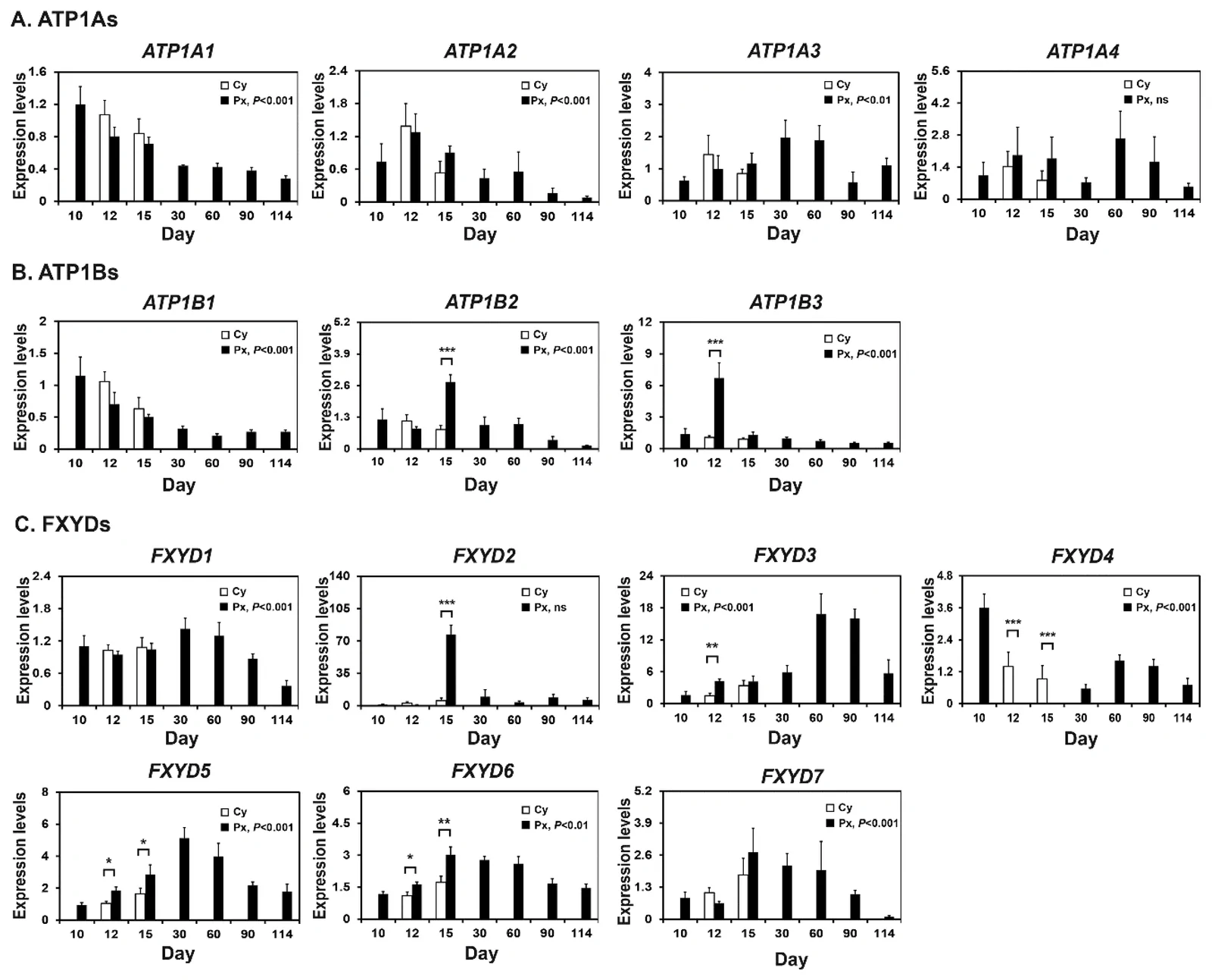
The expression of Na^+^/K^+^-ATPase subunits ATP1As (A), ATP1Bs (B), and FXYDs (C) in the endometrium during the estrous cycle and pregnancy. Endometrial tissue samples from cyclic (Cy) and pregnant gilts (Px) were analyzed by real-time RT-PCR. Data are reported as expression relative to that detected on Day 12 of the estrous cycle after normalization of the transcript amount to endogenous *RPL7*, *UBB*, and *TBP* controls. Data are presented as mean with standard error. * p<0.05, ** p<0.01, *** p<0.001. FXYD, Phe-X-Tyr-Asp; RT-PCR, reverse transcription-polymerase chain reaction; *RPL7*, ribosomal protein L7; *UBB*, ubiquitin B; *TBP*, TATA-binding protein.

To determine the expression of Na^+^/K^+^-ATPase subunits, α (*ATP1A1* - *ATP1A4*), β (*ATP1B1* - *ATP1B3*), and FXYD (*FXYD1* - *FXYD7*) subunits in the endometrium during the estrous cycle and pregnancy in pigs, we measured their relative abundance using real-time RT-PCR analyses (Figure 1). On Days 12 and 15 post-estrus, the expression of *ATP1B2*, *ATP1B3*, *FXYD2*, *FXYD5*, *FXYD6* was affected by day (p<0.001 for *ATP1B2*, *ATP1B3*, *FXYD2*; p<0.05 for *FXYD5*; p<0.01 for *FXYD6*); *ATP1B2*, *ATP1B3*, *FXYD2*, *FXYD3*, *FXYD4*, *FXYD5*, and *FXYD6* by pregnancy status (p<0.001 for *ATP1B2*, *ATP1B3*, *FXYD2*, and *FXYD4*; p<0.05 for *FXYD3* and *FXYD5*; p<0.01 for *FXYD6*); and *ATP1B2*, *ATP1B3*, and *FXYD2* by dayxstatus interaction (p<0.001 for *ATP1B2* and *FXYD2*; p<0.01 for *ATP1B3*) (Figure 1). The expression of *ATP1A1* – *ATP1A4*, *ATP1B1*, and *FXYD1* was not affected by day, pregnancy status, or day x status interaction. The expression of *ATP1B3*, *FXYD3*, *FXYD5*, and *FXYD6* was greater on Day 12 of pregnancy than on Day 12 of the estrous cycle, and the expression of *ATP1B2*, *FXYD5*, and *FXYD6* was greater on Day 15 of the estrous cycle than on Day 15 of pregnancy. The expression of *FXYD4* was greater on Days 12 and 15 of the estrous cycle than on Days 12 and 15 of pregnancy, respectively.

During pregnancy, the expression of *ATP1A1*, *ATP1A2*, *ATP1A3*, *ATP1B1*, *ATP1B2*, *ATP1B3*, *FXYD1*, *FXYD3*, *FXYD4*, *FXYD5*, and *FXYD7*, but not *ATP1A4*, and *FXYD2*, changed in the endometrium (linear effect of day, p<0.001 for *ATP1A1*, *ATP1A2*, *ATP1B1*, *ATP1B2*, and *FXYD4*, p<0.01 for *ATP1B3*; quadratic effect of day, p<0.001 for *FXYD1*, *FXYD3*, *FXYD5*, and *FXYD7*, p<0.01 for *FXYD6*; cubic effect of day, p<0.01 for *ATP1A3*). The expression of *ATP1B3* was greatest on Day 12 and *ATP1B2*, *FXYD2*, and *FXYD6* on Day 15 during pregnancy.

### Expression of Na^+^/K^+^-ATPase subunits in conceptuses during early pregnancy and chorioallantoic tissues during later pregnancy

**Figure 2:**
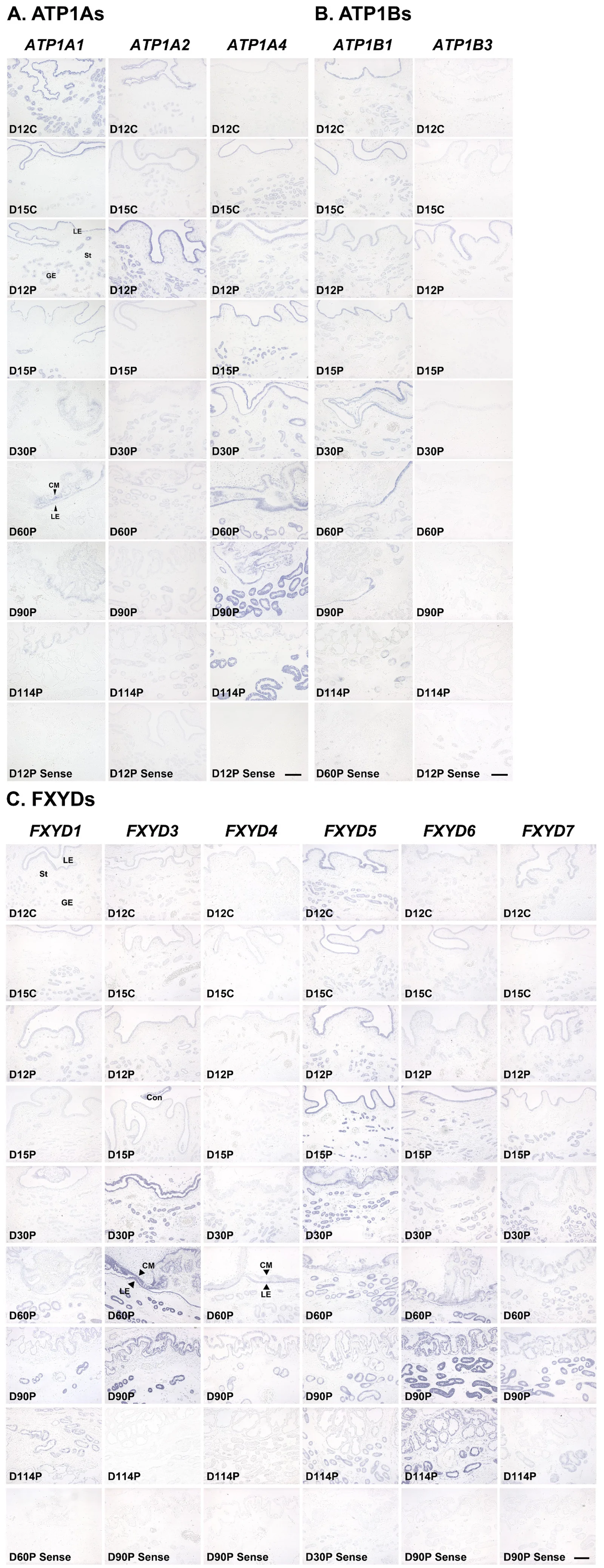
*In situ* hybridization analysis of Na^+^/K^+^-ATPase subunits ATP1As (A), ATP1Bs (B), and FXYDs (C) in the endometrium during the estrous cycle and pregnancy. The expression of ATP1As, ATP1Bs, and FXYDs was localized by *in situ* hybridization. Representative uterine sections from Days 12, 30, 60, or 90 of pregnancy hybridized with digoxigenin-labeled sense probes (sense), respectively, are shown as negative controls for *in situ* hybridization. Bars = 100 μm. LE, luminal epithelium; St, stroma; GE, glandular epithelium; D, day; C, estrous cycle; P, pregnancy; CM, chorioallantoic membrane; FXYD, Phe-X-Tyr-Asp.

To determine whether conceptuses expressed Na^+^/K^+^-ATPase subunit mRNAs during early pregnancy, we performed RT-PCR using cDNAs from conceptuses on Days 12 and 15 of pregnancy. The expression of *ATP1A1*, *ATP1A4*, *ATP1B1* - *ATP1B3*, and *FXYD1* - *FXYD6* was detected in conceptuses on Days 12 and 15 of pregnancy. The expression of *ATP1A3* and *FXYD7* was detected on Day 12, but not on Day 15, of pregnancy, and the expression of *ATP1A2* was not detected on both days of pregnancy (Figure 2A).

To determine whether the expression of Na^+^/K^+^-ATPase subunit mRNAs changed in chorioallantoic tissues during mid- to late pregnancy, we performed real-time RT-PCR analysis using chorioallantoic tissues on Days 30, 60, 90, and 114 of pregnancy. The expression of *ATP1A1* – *ATP1A3*, *ATP1B2*, *ATP1B3*, and *FXYD2* – *FXYD7* changed in chorioallantoic tissues with levels increasing toward term pregnancy (linear effect of day, p<0.05 for *ATP1A1* and *FXYD6*; cubic effect of day, p<0.05 for *ATP1A3*; quadratic effect of day, p<0.01 for *FXYD5*), with levels decreasing toward term pregnancy (cubic effect of day, p<0.05 for *FXYD7*), with biphasic high levels on Day 30 and term pregnancy (quadratic effect of day, p<0.05 for *ATP1A2* and *ATP1B2*, p<0.01 for *FXYD2*), or with biphasic low levels on Day 30 and term pregnancy (cubic effect of day, p<0.05 for *ATP1B3*; quadratic effect of day, p<0.05 for *FXYD3* and *FXYD4*). The expression of *ATP1A4*, *ATP1B1*, *FXYD1* did not change in chorioallantoic tissues during pregnancy (Figures 2B–2D).

### Localization of Na^+^/K^+^-ATPase subunit mRNAs in the endometrium during the estrous cycle and pregnancy in pigs

**Figure 3:**
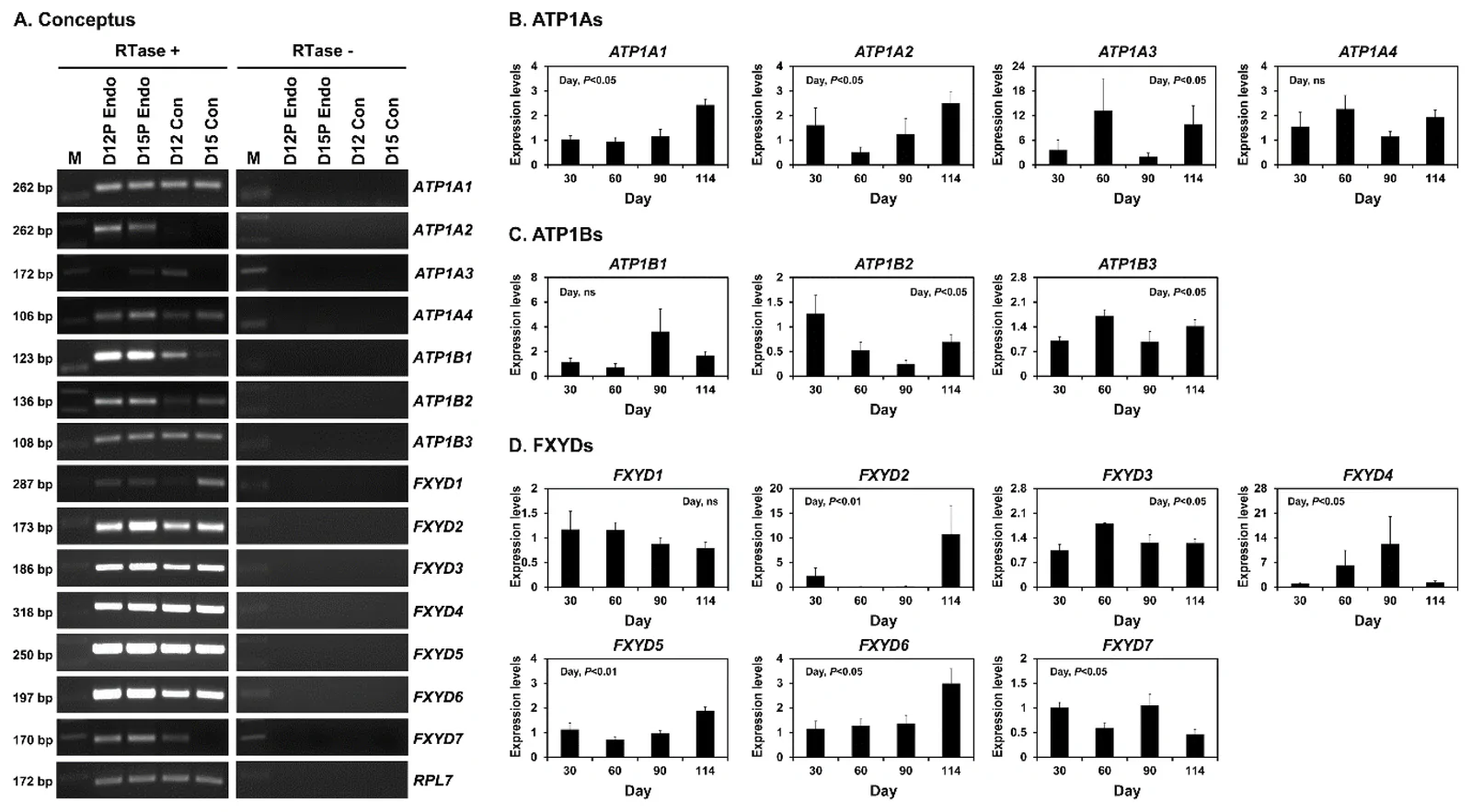
Expression of Na^+^/K^+^-ATPase subunits in conceptuses from Days 12 and 15 of pregnancy and in chorioallantoic tissues during mid to late pregnancy. (A) RT-PCR analysis of Na^+^/K^+^-ATPase subunits in conceptuses on Days 12 and 15 of pregnancy. *RPL7* was used as a positive control for the PCR reaction. (B–D) Real-time RT-PCR analysis of the expression of Na^+^/K^+^-ATPase subunits in chorioallantoic tissues on Days 30, 60, 90, and 114 of pregnancy. Data are reported as expression relative to that detected on Day 30 of pregnancy after normalization of the transcript amount to endogenous *RPL7*, *UBB*, and *TBP* controls, and data are presented as mean with standard error. RTase +/–, with (+) or without (–) reverse transcriptase; M, molecular marker; D12P Endo, endometrium on Day 12 of pregnancy; D15P Endo, endometrium on Day 15 of pregnancy; D12 Con, Day 12 conceptus; D15 Con, Day 15 conceptus; RT-PCR, reverse transcription-polymerase chain reaction; *RPL7*, ribosomal protein L7; *UBB*, ubiquitin B; *TBP*, TATA-binding protein.

To detect the localization of Na^+^/K^+^-ATPase subunit mRNAs in the endometrium during the estrous cycle and pregnancy, we performed *in situ* hybridization (Figure 3). *ATP1A1* mRNA was localized to luminal epithelial (LE) and glandular epithelial (GE) cells but not to stromal cells in the endometrium, with stronger signal intensity in epithelial cells during the estrous cycle and early pregnancy than mid- to late stage of pregnancy. *ATP1A1* mRNA was also localized to CE cells in chorioallantoic membrane in areolae regions during mid- to late pregnancy. *ATP1A2* mRNA was localized to LE, GE, and stromal cells in the endometrium, with strong signal intensity during the estrous cycle and early pregnancy. *ATP1A4* mRNA was detected in LE, GE, and stromal cells in the endometrium and CE and stromal cells in chorioallantoic tissues throughout pregnancy (Figure 3A). *ATP1B1* mRNA was also detected in LE, GE, and stromal cells in the endometrium and CE and stromal cells in chorioallantoic tissues throughout pregnancy, and strong signal intensity was detected in endometrial LE cells in areolae regions during mid- to late pregnancy. *ATP1B3* mRNA was localized primarily to LE cells in the endometrium on Day 12 of pregnancy (Figure 3B). *FXYD1*, *FXYD3*, *FXYD5*, and *FXYD6* mRNAs were localized to LE, GE, and stromal cells in the endometrium during the estrous cycle and pregnancy and to CE and stromal cells in chorioallantoic tissues during pregnancy. *FXYD4*, and *FXYD7* mRNAs were localized primarily to LE and GE cells in the endometrium during the estrous cycle and pregnancy and CE cells in chorioallantoic tissues during pregnancy (Figure 3C). The expression of *ATP1A3*, *ATP1B2*, and *FXYD2* was not localized in the endometrium due to weak *in situ* hybridization signal intensity.

### Effects of steroid hormones, interleukin-1β, and interferon-γ on the expression of Na^+^/K^+^-ATPase subunits in endometrial tissues

**Figure 4:**
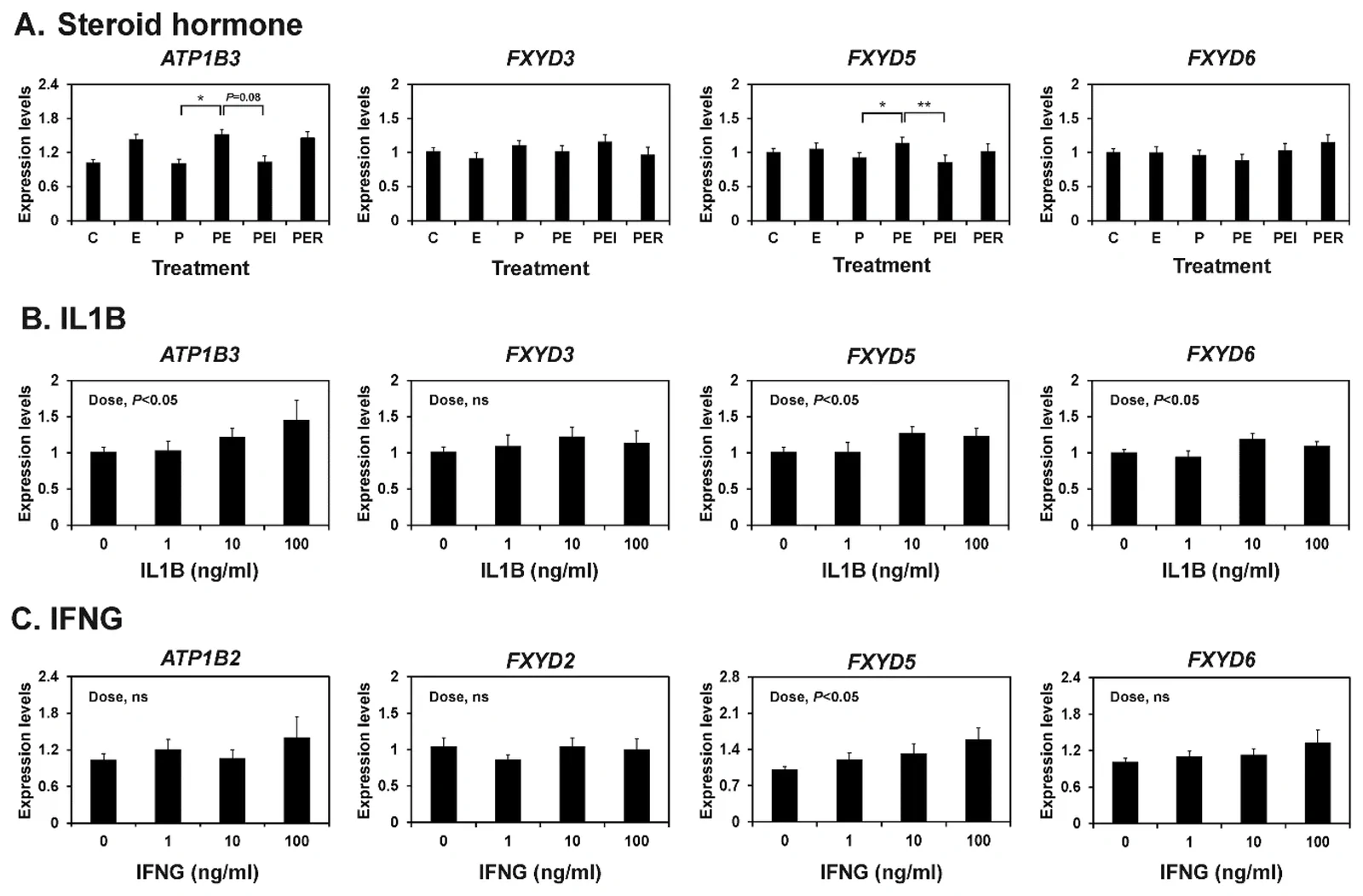
Effects of steroid hormones (A), IL1B (B), and IFNG (C) on the expression of Na^+^/K^+^-ATPase subunits in endometrial explant cultures. Endometrial explants from gilts on Day 12 of the estrous cycle were cultured with steroid hormones (A), with increasing doses of IL1B (0, 1, 10, and 100 ng/mL) in the presence of E2 (10 ng/mL) and P4 (30 ng/mL) (B), or with increasing doses of IFNG (0, 1, 10, and 100 ng/mL) in the presence of E2 (10 ng/mL) and P4 (30 ng/mL) (C). The abundance of mRNA expression, as determined by real-time RT-PCR analyses, is relative to that of Na^+^/K^+^-ATPase subunit mRNAs in the control group of endometrial explants after normalization of the transcript amounts to *RPL7*, *UBB*, and *TBP* mRNAs. Data are presented as mean with standard error. These treatments were performed in triplicate using tissues obtained from each of three gilts. * p<0.05, ** p<0.01. C, control; E, estradiol-17β (E2), P, progesterone (P4); PE, P4+E2; PEI, P4+E2+ICI (ICI, an estrogen receptor antagonist); PER, P4+E2+RU (RU; a progesterone receptor antagonist); IL1B, interleukin-1β; IFNG, interferon-γ; RT-PCR, reverse transcription-polymerase chain reaction; *RPL7*, ribosomal protein L7; *UBB*, ubiquitin B; *TBP*, TATA-binding protein.

Given that the expression of *ATP1B3*, *FXYD3*, *FXYD5*, and *FXYD6* mRNAs in the endometrium was greater on Day 12 of pregnancy than on Day 12 of the estrous cycle and the expression of *ATP1B2*, *FXYD2*, *FXYD5*, and *FXYD6* mRNAs was greater on Day 15 of pregnancy than Day 15 of the estrous cycle (Figure 1), and that porcine conceptuses secrete estrogen and IL1B into the uterine lumen on Day 12 and IFNs on Day 15, respectively [1]), we hypothesized that the steroid hormones estrogen and progesterone and the cytokines IL1B and IFNG affect the expression of Na^+^/K^+^-ATPase subunits in the endometrium during early pregnancy. Thus, we determined whether E2, P4, and IL1B affected the expressions of *ATP1B3*, *FXYD3*, *FXYD5*, and *FXYD6* and whether IFNG affected the expressions of *ATP1B2*, *FXYD2*, *FXYD5*, and *FXYD6* in endometrial tissue explants (Figure 4).

The expression of *ATP1B3* and *FXYD5*, but not *FXYD3* and *FXYD6*, was increased by E2 in the presence of P4 (P4 vs P4+E2, p<0.05 for *ATP1B3* and *FXYD5*) and the E2-induced increases in *ATP1B3* and *FXYD5* expression were inhibited by ICI, an estrogen receptor antagonist in endometrial explant tissues (P4+E2 vs. P4+E2+ICI, p = 0.0798 for *ATP1B3*, p<0.01 for *FXYD5*) (Figure 4A). The increasing doses of IL1B increased the expression of *ATP1B3*, *FXYD5*, and *FXYD6*, but not *FXYD3* (linear effect of dose, p<0.05 for *ATP1B3*; quadratic effect of dose, p<0.05 for *FXYD5* and *FXYD6*) (Figure 4B). In addition, the increasing doses of IFNG increased the expression of *FXYD5* (linear effect of dose, p<0.05), but not *ATP1B2*, *FXYD2*, and *FXYD6* in endometrial explant tissues (Figure 4C).

### Effects of intracellular calcium ions on the expression of Na^+^/K^+^-ATPase subunits in endometrial tissues

**Figure 5:**
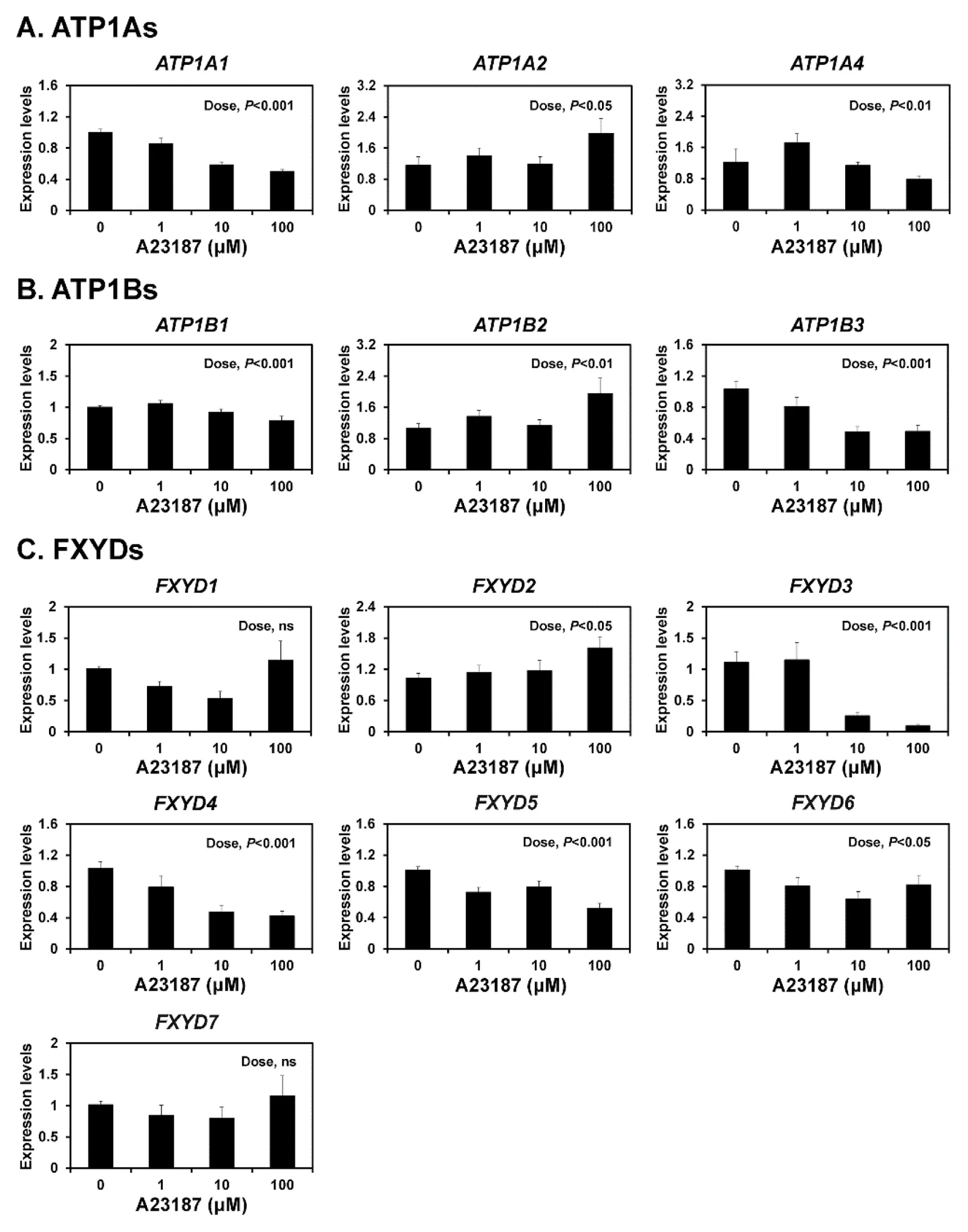
Effects of calcium ions on the expression of Na^+^/K^+^-ATPase subunits ATP1As (A), ATP1Bs (B), and FXYDs (C) in endometrial explant cultures. Endometrial explants from gilts on Day 12 of the estrous cycle were cultured with increasing doses of A23187 (a calcium ionophore; 0, 1, 10, and 100 μM) in the presence of E2 (10 ng/mL) and P4 (30 ng/mL). The abundance of mRNA expression, as determined by real-time RT-PCR analyses, is relative to that of Na^+^/K^+^-ATPase subunit mRNAs in the control group of endometrial explants after normalization of the transcript amounts to *RPL7*, *UBB*, and *TBP* mRNAs. Data are presented as mean with standard error. These treatments were performed in triplicate using tissues obtained from each of three gilts. FXYD, Phe-X-Tyr-Asp; RT-PCR, reverse transcription-polymerase chain reaction; *RPL7*, ribosomal protein L7; *UBB*, ubiquitin B; *TBP*, TATA-binding protein.

Implanting porcine conceptuses increase the expression of calcium transporters in endometrial epithelial cells and the secretion and absorption of calcium ions at the maternal-conceptus interface, and calcium ions induce many endometrial genes related to conceptus implantation [10,31–33]. Thus, we hypothesized that calcium ions affect the expression of Na^+^/K^+^-ATPase subunits on Day 12 of pregnancy in the endometrium, and further determined whether A23187, a calcium ionophore, affected the expression of Na^+^/K^+^-ATPase subunits in endometrial tissue explants (Figure 5). Increasing doses of A23187 increased the expressions of *ATP1A2*, *ATP1B2*, and *FXYD2* (linear effect of dose, p<0.05 for *ATP1A2* and *FXYD2*, p<0.01 for *ATP1B2*), whereas A23187 decreased the expressions of *ATP1A1*, *ATP1A4*, *ATP1B1*, *ATP1B3*, *FXYD3*, *FXYD4*, *FXYD5*, and *FXYD6* in endometrial tissues (linear effect of dose, p<0.01 for *ATP1A4*, p<0.001 for *ATP1A1*, *ATP1B1*, *FXYD3*, *FXYD5*, p = 0.001 for *FXYD4*; quadratic effect of dose, p<0.05 for *FXYD6*, p<0.001 for *ATP1B3*) (Figures 5A–5C). The expression of *FXYD1* and *FXYD7* was not affected by A23187 (Figures 5A–5C).

## DISCUSSION

**Figure 6:**
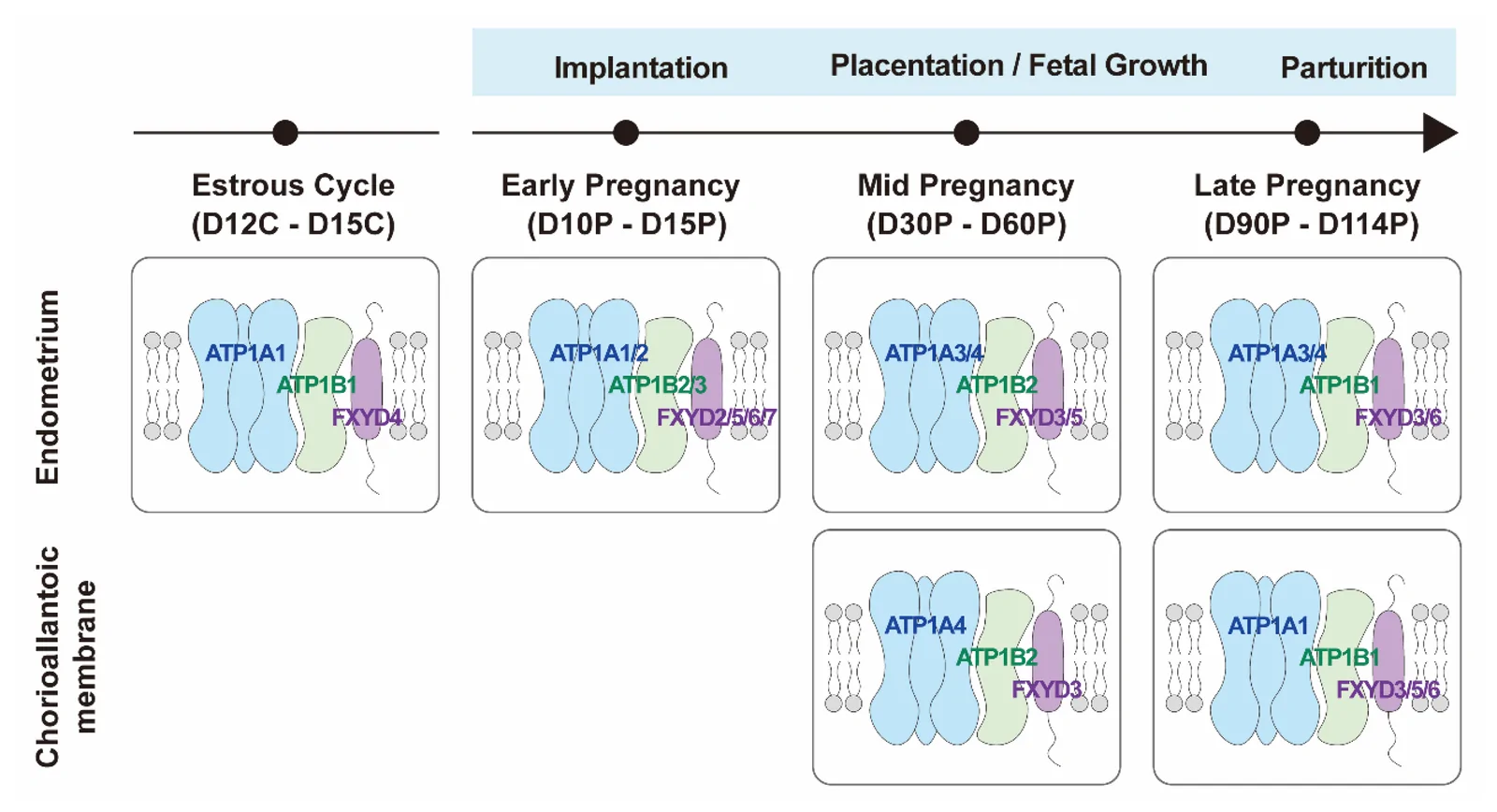
Summary of the expression of Na^+^/K^+^-ATPase subunits at the maternal-conceptus interface in pigs. Developmental processes that occur in the female reproductive tract to corresponding days of pregnancy and the period of the greatest expression of each combination of Na^+^/K^+^-ATPase subunits in the endometrium and chorioallantoic tissues during the stage of pregnancy are indicated. D, day; C, estrous cycle; P, pregnancy.

The novel findings of this study in pigs are that: (1) Na^+^/K^+^-ATPase subunits, ATP1As, ATP1Bs, and FXYDs, are expressed in the endometrium during the estrous cycle and pregnancy in a pregnancy status- and stage-dependent manner; (2) conceptuses on Days 12 and 15 of pregnancy and chorioallantoic tissues from Day 30 to term pregnancy express Na^+^/K^+^-ATPase subunits; (3) Na^+^/K^+^-ATPase subunits are localized primarily to endometrial epithelial and CE cells; (4) E2 increases the expression of *ATP1B3* and *FXYD5*, IL1B increases the expressions of *ATP1B3*, *FXYD5*, and *FXYD6*, and IFNG increases the expression of *FXYD5* in endometrial explant tissues; and (5) A23187 increases the expression of *ATP1A2*, *ATP1B2*, and *FXYD2*, and decreases the expression of *ATP1A1*, *ATP1A4*, *ATP1B1*, *ATP1B3*, *FXYD3*, *FXYD4*, *FXYD5*, and *FXYD6* in endometrial explant tissues (Figure 6). To the best of our knowledge, this is the first report to comprehensively characterize the expression of Na^+^/K^+^-ATPase subunits at the maternal–conceptus interface in pigs.

The endometrium is a highly dynamic tissue that undergoes proliferation, differentiation, secretion, and remodeling, processes tightly regulated by a variety of factors, including steroid hormones, cytokines, and nutrients during the estrous cycle and pregnancy [1]. Appropriate regulation of ion transport in endometrial epithelial cells is critical for cell adhesion and uterine secretions of histotrophs to create an environment compatible with embryo survival and implantation [1]. Na^+^/K^+^-ATPase-mediated Na^+^ transport across the luminal epithelium is a key determinant of uterine fluid composition [34]. In humans and rodents, Na^+^/K^+^-ATPase subunits, ATP1As, ATP1Bs, and FXYDs, are expressed in the endometrium at different levels during pregnancy [22,23]. Results of the present study in pigs showed that Na^+^/K^+^-ATPase subunits were expressed and regulated in a pregnancy status- and stage-dependent manner in the endometrium. In particular, the expression of *ATP1A1*, *ATP1A2*, *ATP1B2*, *ATP1B3*, *FXYD2*, and *FXYD3* in the endometrium was greater during the implantation period than other stages of pregnancy, whereas the expression of *FXYD4* was low during the implantation period. In addition, the expression of *FXYD3* was greater on Day 12 of pregnancy than on Day 12 of the estrous cycle and the expression of *FXYD5* and *FXYD6* in the endometrium was greater on Days 12 and 15 of pregnancy than on Days 12 and 15 of the estrous cycle, respectively. These results suggest that regulating endometrial Na^+^/K^+^-ATPase subunit expression is essential for conceptus-endometrium communication and implantation, thereby modulating the flux of Na^+^/K^+^ ions and transport of nutrients, including glucose, amino acids, and calcium, as well as maintaining the membrane potential of endometrial epithelial cells.

Besides its importance in trophectoderm cell differentiation in rodent blastocyst [35], the expression of Na^+^/K^+^-ATPase plays essential roles in decidualization and differentiation of cytotrophoblast cells in humans, and inappropriate expression is associated with preeclampsia and spontaneous labor [36–38]. Results of the present study in pigs showed that Na^+^/K^+^-ATPase subunits were expressed in implanting conceptuses during early pregnancy and chorioallantoic tissues during mid- to late pregnancy. In conceptuses on Days 12 and 15 of pregnancy, the expression of most Na^+^/K^+^-ATPase subunits studied, except *ATP1A2* on both days and *ATP1A3* and *FXYD7* on Day 15, was detected. In chorioallantoic tissues from Day 30 of pregnancy to term, the expression of most of Na^+^/K^+^-ATPase subunits studied, except *ATP1A4*, *ATP1B1*, and *FXYD1*, changed in a stage-specific manner. These results in pigs suggest that the expression of Na^+^/K^+^-ATPase subunits may be crucial for trophoblast differentiation and placental functions such as transport of nutrients and initiation of parturition.

In the human and rodent endometrium, Na^+^/K^+^-ATPase subunits, ATP1As, ATP1Bs, and FXYDs, are localized to epithelial and stromal cells in the endometrium during pregnancy [22,23]. In human placentas, Na^+^/K^+^-ATPase is also distributed to the syncytiotrophoblast layer, which absorbs various nutrients from the maternal blood, and IUGR is associated with the reduced expression of Na^+^/K^+^-ATPase in syncytiotrophoblast [16,17]. The results of the present study in pigs showed that the expression of Na^+^/K^+^-ATPase subunits, ATP1As, ATP1Bs, and FXYDs, was localized to LE, GE, and stromal cells in the endometrium during the estrous cycle and pregnancy, as well as to CE and stromal cells in chorioallantoic membranes during pregnancy. In particular, the expression signals of the Na^+^/K^+^-ATPase subunits varied depending on the isoforms and pregnancy stages. Specifically, the expression signals for *ATP1A1*, *ATP1A2*, *ATP1B1*, and *ATP1B3* were stronger during early pregnancy than during mid- to late pregnancy and the expression of *ATP1A1*, *ATP1B1*, and *ATP1B3* was epithelial cell-specific during early pregnancy. These results suggest that the endometrium undergoes cell-specific remodeling of Na^+^/K^+^-ATPase subunit isoform repertoire during pregnancy. Such distinct and coordinated changes may play critical roles in ionic and fluid homeostasis, uterine secretion, and conceptus/fetal support throughout pregnancy.

In diverse tissues and cells, the expression of Na^+^/K^+^-ATPase subunits, ATP1As, ATP1Bs, and FXYDs, is regulated by a variety of factors, including hormones (aldosterone, cortisol, corticosterone, insulin, and progesterone), cytokine (IL2), and growth factors (epidermal growth factor, fibroblast growth factor 2, and transforming growth factor-β) [39]. Our results showed that the expression of *ATP1B3*, *FXYD3*, *FXYD5*, and *FXYD6* mRNAs in the endometrium was greater on Day 12 of pregnancy than on Day 12 of the estrous cycle and the expressions of *ATP1B2*, *FXYD2*, *FXYD5*, and *FXYD6* mRNAs were greater on Day 15 of pregnancy than Day 15 of the estrous cycle. During the implantation period, the porcine conceptus secretes estrogen and IL1B on Day 12 of pregnancy and IFNs on Days 14 and 16 into the uterine lumen, and these conceptus-derived hormones and cytokines, as well as progesterone of ovarian origin, induce the expression of many endometrial genes [1,40]. Thus, we speculated that these hormones and cytokines are responsible for the expression of Na^+^/K^+^-ATPase subunits in the endometrium. Indeed, E2 increased the expressions of *ATP1B3* and *FXYD5*, IL1B increased the expression of *ATP1B3*, *FXYD5*, and *FXYD6*, and IFNG increased the expression of *FXYD5* in endometrial tissue explants. These data suggest that conceptus-derived factors regulate the endometrial expression of Na^+^/K^+^-ATPase subunits, thereby modulating the flux of Na^+^/K^+^ ions and the membrane potential of endometrial cells, specifically epithelial cells, for the establishment of pregnancy in pigs.

In addition to hormones and cytokines, elevated calcium ions also increase the expression of *Atp1a1* and *Atp1b1* in rat kidney [41]. Calcium ions play crucial roles in cell-to-cell interactions between the endometrial epithelial cells and conceptus trophectoderm, inducing the expression of numerous endometrial genes, while active influx and efflux of these ions occur across the epithelial layer during the implantation period [10,31–33]. Thus, we postulated that calcium ions affect the expression of Na^+^/K^+^-ATPase subunits during early pregnancy. Our results showed that A23187, a calcium ionophore, increased the expression of *ATP1A2*, *ATP1B2*, and *FXYD2*, whereas it decreased the expression of *ATP1A1*, *ATP1A4*, *ATP1B1*, *ATP1B3*, *FXYD3*, *FXYD4*, *FXYD5*, and *FXYD6* in endometrial tissues. These results suggest that calcium ions play important roles in regulating the expression of Na^+^/K^+^-ATPase subunits, thereby controlling the Na^+^/K^+^-ATPase activity for Na^+^/K^+^ flux across the endometrial epithelial layer to establish endometrial receptivity for conceptus trophectoderm during the peri-implantation period in pigs.

Na^+^/K^+^-ATPase not only maintains sodium and potassium ion concentrations to preserve cell membrane potential, but also facilitates the transport of various nutrients, such as glucose, amino acids, and calcium ions [13,14]. Genome-wide analysis of placental efficiency shows that genes related to nutrient transport and metabolic homeostasis are closely associated with the increased litter size in pigs [42]. Thus, our findings suggest that the stage-specific differential expression of ATP1As, ATP1Bs, and FXYDs in the endometrium and chorioallantoic membrane play critical roles in tightly regulating membrane potential and nutrient transport at the maternal-conceptus interface in pigs. In addition, Na^+^/K^+^-ATPase is involved in cell adhesion and migration by interacting with E-cadherin [43], which is expressed in porcine endometrial epithelial cells and implanting conceptuses during early pregnancy [44,45]. This suggests that Na^+^/K^+^-ATPase may also facilitate cell-cell adhesion between the endometrial epithelial cells and the conceptus trophectoderm by regulating epithelial junctional organization, polarity, or adhesion-related remodeling during the implantation period in pigs. Nevertheless, the detailed molecular and functional mechanisms underlying Na^+^/K^+^-ATPase activity in nutrient transport and cell adhesion remain to be determined in future studies.

## CONCLUSION

In conclusion, the results of this study in pigs demonstrate that Na^+^/K^+^-ATPase subunits are expressed in endometrial, conceptus, and chorioallantoic tissues in a pregnancy-stage-specific manner. Conceptus-derived estrogen, IL1B, and IFNG, alongside calcium ions, regulate the expression of multiple Na^+^/K^+^-ATPase subunits in endometrial tissues during the implantation period. These results suggest that Na^+^/K^+^-ATPase subunits, dynamically regulated by conceptus signals and pregnancy stage, play critical roles in maintaining membrane potential by controlling Na^+^/K^+^ flux in endometrial and conceptus cells. Furthermore, they may modulate nutrient transport through interactions with diverse nutrient transporters, thereby facilitating the successful establishment and maintenance of pregnancy in pigs.

## Figures and Tables

**Table 1:** Summary of primer sequences for real-time RT-PCR and RT-PCR and expected product sizes

Primer	Sequence of forward (F) and reverse (R) primers (5′ → 3′)	Annealing temperature (°C)	Product size (bp)	GenBank accession no.
For real-time PCR and RT-PCR				
*ATP1A1*	F: GAAACCCCAAGACAGACAAA	60	116	NM_214249.1
	R: CTCAGCCAGGATCACAAAGT			
*ATP1A2*	F: GGATGACCACAAGCTGTCCT	60	262	NM_000702.3
	R: GTCATTGGATGGTTCATCCTC			
*ATP1A3*	F: TGGCAGAAAACGGCTTCTTG	60	172	NM_001171753.1
	R: TGCACAACCACTATGCTCAC			
*ATP1A4*	F: CAGAATACCAGCCCAAGTCC	60	106	XM_001928979.2
	R: GGACCACTTCTTCCTTCAGC			
*ATP1B1*	F: AAACCTAAGCCTCCCAAGAA	60	123	NM_001001542.1
	R: CATGGTTCCAACTTTCTCCT			
*ATP1B2*	F: TACCTCGTCTTCTATGGCTTCC	60	136	XM_003131945.2
	R: CAGTCTTAGGACGAATCATCAGG			
*ATP1B3*	F: AATGTGGGTTATGCTTCAGACC	60	108	XM_003132450.3
	R: TAATCCAACGCTGACACTGG			
*FXYD1*	F: CTCAGCCACATCTTGGTTCTC	60	287	XM_003355850.2
	R: CATAGCGCCAGGTGTTTCTAC			
*FXYD2*	F: GTTGTCGACGGACGATGG	60	173	NM_001014427.1
	R: TAATAGGCCGGTGCTTCTTG			
*FXYD3*	F: CTCTGCTTTGGATGCCAACG	60	158	NM_214208.2
	R: TCTGGCTGAACTTGCATTTGC			
*FXYD4*	F: AACTGTGTCATGGAGGGTGTG	60	318	XM_003133041.3
	R: ATGGAAACTGGTACAGGGAGTG			
*FXYD5*	F: ATTGCCTGTGTCTCATCATCAC	60	250	XM_003355854.1
	R: CCCAGATCTGTTGTTAGAAGCAC			
*FXYD6*	F: TGAGCAGGAGAAAGAAAAGGAC	60	197	NM_001034399.1
	R: CATTTGCAGTGACGAGGTTC			
*FXYD7*	F: TGAGGAACCTGACCCTTTT	60	174	NM_022006.1
	R: GCTCAGACTTACAGGATTTGC			
*RPL7*	F: AAGCCAAGCACTATCACAAGGAATACA	60	172	NM_0011132176
	R: TGCAACACCTTTCTGACCTTTGG			
*UBB*	F: GCATTGTTGGCGGTTTCG	60	81	NM_001105309.1
	R: AGACGCTGTGAAGCCAATCA			
*TBP*	F: AACAGTTCAGTAGTTATGAGCCAGA	60	262	DQ845178.1
	R: AGATGTTCTCAAACGCTTCG			
For *in situ* hybridization				
*ATP1A1*	F: ATTGCCTACACCCTCACCAG	60	490	NM_214249.1
	R: TCTTGCAGATGACCAAGTCG			
*ATP1A2*	F: CTGTCACGGTGTGTTTGACC	60	830	NM_000702.3
	R: CACCATGATCACCTTGATGC			
*ATP1A4*	F: CAGGGAAAGAAAGATGAAGAGG	60	233	XM_001928979.2
	R: GCTGTTTACAGAATTTGACCCATT			
*ATP1B1*	F: CTGGCATCTTCATTGGAACC	60	685	NM_001001542.1
	R: TCTATGCGGATTTCAGTGTCC			
*ATP1B3*	F: TAACATCGCAATGTGCTTCC	60	520	XM_003132450.3
	R: TCCCGCCCTAAAAATAATCC			
*FXYD1*	F: GCCGGGAGCCTATTTTGG	60	506	XM_003355850.2
	R: GAGAGAAAAGCACGTTTTATTGG			
*FXYD2*	F: GTTGTCGACGGACGATGG	60	173	NM_001014427.1
	R: TAATAGGCCGGTGCTTCTTG			
*FXYD3*	F: GAGGACGGATCAGCAGAAAG	60	186	NM_214208.1
	R: CGGGAGGCAAGATCATTTTA			
*FXYD4*	F: CCCATTTGCCAATAAAGACG	60	120	XM_003133041.3
	R: CATTTGCCACTCAGAACTGC			
*FXYD5*	F: ATTGCCTGTGTCTCATCATCAC	60	250	XM_003355854.1
	R: CCCAGATCTGTTGTTAGAAGCAC			
*FXYD6*	F: GAGGTGTTGCTGATGTTC	60	258	NM_001034399.1
	R: CATTTGCAGTGACGAGGTTC			
*FXYD7*	F: TGAGGAACCTGACCCTTTT	60	166	NM_022007.1
	R: AAGCTCCGACTTACAGGATT			

RT-PCR, reverse transcription polymerase chain reaction; *ATP1A*, Na^+^/K^+^-ATPase α subunit; *ATP1B*, Na^+^/K^+^-ATPase β subunit; *FXYD*, Phe-X-Tyr-Asp (FXYD) protein subunit; *RPL7*, ribosomal protein L7; *UBB*, ubiquitin B; *TBP*, TATA binding protein.
